# Drug Induced Steatohepatitis: An Uncommon Culprit of a Common Disease

**DOI:** 10.1155/2015/168905

**Published:** 2015-07-26

**Authors:** Liane Rabinowich, Oren Shibolet

**Affiliations:** Liver Unit, Department of Gastroenterology, Tel Aviv Medical Center, 6423906 Tel Aviv, Israel

## Abstract

Nonalcoholic fatty liver disease (NAFLD) is a leading cause of liver disease in developed countries. Its frequency is increasing in the general population mostly due to the widespread occurrence of obesity and the metabolic syndrome. Although drugs and dietary supplements are viewed as a major cause of acute liver injury, drug induced steatosis and steatohepatitis are considered a rare form of drug induced liver injury (DILI). The complex mechanism leading to hepatic steatosis caused by commonly used drugs such as amiodarone, methotrexate, tamoxifen, valproic acid, glucocorticoids, and others is not fully understood. It relates not only to induction of the metabolic syndrome by some drugs but also to their impact on important molecular pathways including increased hepatocytes lipogenesis, decreased secretion of fatty acids, and interruption of mitochondrial *β*-oxidation as well as altered expression of genes responsible for drug metabolism. Better familiarity with this type of liver injury is important for early recognition of drug hepatotoxicity and crucial for preventing severe forms of liver injury and cirrhosis. Moreover, understanding the mechanisms leading to drug induced hepatic steatosis may provide much needed clues to the mechanism and potential prevention of the more common form of metabolic steatohepatitis.

## 1. Introduction

Drug induced liver injury (DILI) is a leading cause of acute liver failure and transplantation in western countries; and although rare, represents a serious clinical problem due to its unpredictable nature and possibly fatal course [1]. The liver plays a central role in drug metabolism and clearance and is therefore susceptible to DILI. Liver injury remains the most common cause of clinical trial termination and a main cause of postmarketing drug withdrawals. DILI may occur in a dose-dependent way, yet the majority of cases are idiosyncratic and dose independent. The precise incidence of DILI is difficult to determine and is estimated to range from 1/10,000 to 1/1,000,000 prescription-years [2]. A recent population based study from Iceland demonstrated an annual incidence rate of 19 cases per 100,000 inhabitants, with a single prescription medication being the cause for DILI in 75% of the cases [3].

Nonalcoholic fatty liver disease (NAFLD) is recognized as a leading cause of liver disease in the western world and has increased in frequency over recent decades [4]. The prevalence of NAFLD ranges from 20 to 30% in the general population, depending on diagnostic criteria and the specific definition applied, with rates exceeding 50% in diabetic and obese patients [4–6]. NAFLD includes a spectrum of liver diseases ranging from simple hepatic steatosis to nonalcoholic steatohepatitis (NASH), liver cirrhosis, and hepatocellular carcinoma (HCC) [7]. Drug induced steatosis (DIS) or steatohepatitis (DISH) is a rare form of DILI with fewer than 2% of all cases of NASH attributed to drugs [8]. Grieco et al. divided drugs capable of inducing steatosis and steatohepatitis into three groups: drugs that induce metabolic changes and can precipitate latent NASH (e.g., tamoxifen), drugs that cause steatosis and steatohepatitis independently (e.g., amiodarone, perhexiline maleate), and drugs that induce sporadic events of steatosis/steatohepatitis (e.g., carbamazepine) [8].

## 2. Diagnosis of DILI

The diagnosis of DILI is challenging largely due to the fact that it remains a clinical diagnosis of exclusion. A reliable diagnosis requires demonstration of close correlation between the patient history and clinical, laboratory, and histological data. The initial presentation is typically elevated liver enzymes with or without jaundice, with a pattern that may be hepatocellular, cholestatic, or mixed. The differential diagnosis is wide including viral, autoimmune, metabolic, and genetic disorders, and clinical presentation may range from complete lack of symptoms to mild constitutional signs (fever, fatigue, and right upper quadrant pain) to severe liver injury and even fulminant hepatitis. The ability to reach a definitive diagnosis is further complicated by the lack of objective diagnostic tests and the extensive list of drugs and herbal or dietary supplements that are recognized as potential causes of DILI. Consequently, a meticulous patient history and appropriate laboratory and imaging studies are required in order to exclude other etiologies before a reliable diagnosis of DILI can be established. A crucial element in establishing a diagnosis of DILI is the temporal relationship between drug exposure to development of liver injury and resolution following discontinuation of the drug. The manifestations of liver toxicity typically occur within days or weeks but, in some cases, may appear after months or even years of use and may even occur after treatment with the drug has been stopped. Moreover, liver enzyme elevations can persist for several months after the drug was discontinued.

Several scoring systems were developed in an attempt to standardize the process of causality assessment in DILI such as The Council for International Organizations of Medical Sciences/Roussel Uclaf Causality Assessment Method (CIOMS/RUCAM) scale [9, 10], the Naranjo Adverse Drug Reactions Probability Scale (NADRPS) [11] and the Maria and Victorino method [12]. The RUCAM scale is the most commonly used and consists of information regarding the type of enzymatic injury, known risk factors (e.g., age, alcohol use, and pregnancy), exclusion of other causes of liver injury (e.g., viral hepatitis, ischemia, underlying liver disease), and a timeline consisting of disease onset, improvement after withholding the drug, and rechallenge [9]. It is worth noting that, on the basis of the RUCAM scale, DILI is most likely to develop within 90 days of initiating treatment and show improvement within 30 days of discontinuation; this is frequently not the case in DIS/DISH as we refer to later in regard to specific drugs.

## 3. Histological Patterns of NAFLD, NASH, DIS, and DISH

The principal histological feature of hepatic steatosis is defined as deposition of fat, principally triglycerides, within the hepatocyte. A minimum of 5% steatosis is required for histological definition of NAFLD. NASH is defined as the presence of hepatic steatosis along with inflammation and hepatocyte injury (ballooning), with or without fibrosis [13]. Several pathological classifications have been proposed for NAFLD: Matteoni's classification, Brunt's classification, and the NALFD activity score (NAS) [14–16].

Liver biopsy is not considered a routine part of the clinical evaluation in cases of suspected DILI or NASH. However, it may offer further information in an attempt to evaluate a patient's liver injury in clinically complex situations, as well as defining the degree of injury. A correlation between the histological pattern and history of suspected drug use may strengthen the diagnosis of DILI based on formally recognized injury patterns and may also help exclude other potential etiologies of liver injury. The five most common histological patterns observed in 83% of DILI cases are acute and chronic hepatitis, acute and chronic cholestasis, and mixed cholestatic hepatitis [17]. Steatohepatitis is a relatively rare form of DILI; data from the Spanish group for the study of drug induced liver disease noted that only 2 out of the 110 cases with available liver histology showed a predominant pattern defined as steatosis [18]; however, more resent data from the Drug Induced Liver Injury Network (DILIN) indicated that although this is rarely described as the dominant pattern, 26% of cases showed some degree of steatosis, with macrovesicular steatosis as the dominant pattern in over 70% of the cases [17].

Many drugs may cause steatosis or steatohepatitis with pathological features resembling those of alcoholic fatty liver disease or NAFLD. The steatosis caused by drugs can be further characterized based on the predominant steatotic feature (Table 1).

*Macrovesicular steatosis* is described as the presence of small to large lipid droplets in the hepatocyte cytoplasm, with peripheral displacement of the cell nucleus [19–21]. This form of liver injury is often reversible; nevertheless, over time it may evolve to steatohepatitis and even cirrhosis. Macrovesicular steatosis is associated with excess alcohol exposure and with treatment with glucocorticoids, total parenteral nutrition (TPN) [22], methotrexate (MTX), and amiodarone. Chemotherapy associated steatosis or seatohepatitis (CASH) related to 5-fluorouracil (5-FU), tamoxifen, irinotecan (IRI), cisplatin, and asparaginase [23] may also cause macrovesicular steatosis.

*Steatohepatitis* is characterized by steatosis, necroinflammation, hepatocellular ballooning, with or without Mallory hyaline bodies, and in some cases perisinusoidal fibrosis. Drugs associated with steatohepatitis are amiodarone, MTX, tamoxifen, and IRI.

*Microvesicular steatosis* presents as accumulation of numerous very small droplets in the hepatocyte cytoplasm, without peripheral displacement of the nucleus [20]. This is a more severe form of liver injury, usually associated with mitochondrial dysfunction and when extensive or long lasting may be life threatening. Drugs associated with microvesicular steatosis include valproic acid (VPA), tetracycline (intravenous administration of high doses) [24], aspirin (Reye's syndrome) [25], nucleoside reverse transcriptase inhibitors (NRTI), glucocoiticoids [26], nonsteroidal anti-inflammatory drugs (NSAIDS) [27], and cocaine [28].

## 4. Mechanism of Drug Induced Steatosis and Steatohepatitis

**Figure 1:**
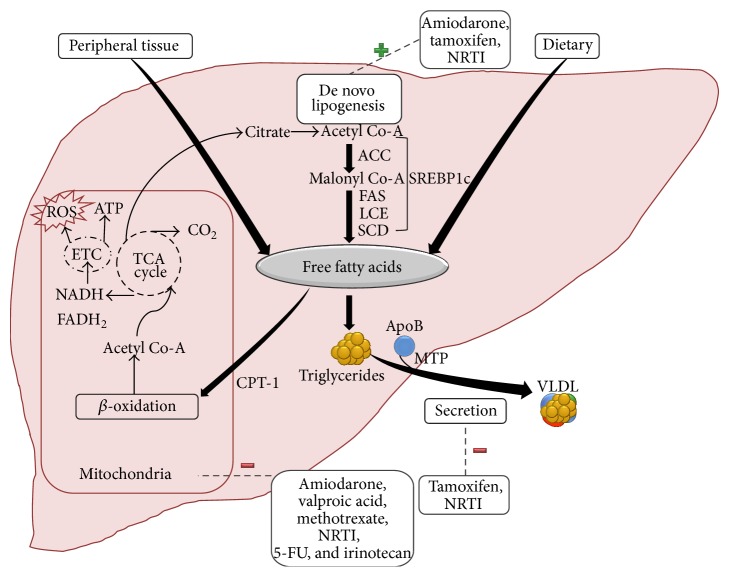
Mechanism of drug induced hepatic steatosis/steatohepatitis. ATP-citrate lyase (ACL), acetyl-CoA carboxylase (ACC), fatty acid synthase (FAS), long-chain fatty acyl elongase (LCE), stearoyl-CoA desaturase (SCD), sterol regulatory element-binding protein-1c (SREBP-1c), apolipoprotein B (ApoB), triglyceride transfer protein (MTP), very-low-density lipoprotein (VLDL), carnitine-palmitoyl-transferase I (CPT I), tricarboxylic acid cycle (TCA cycle), electron transport chain (ETC), reactive oxygen species (ROS), nucleoside reverse transcriptase inhibitors (NRTI), valproic acid (VPA), methotrexate (MTX), 5-fluorouracil (5-FU), and irinotecan (IRI).

Hepatic steatosis is characterized by accumulation of intrahepatocytes triglycerides-esters derived from glycerol and free fatty acids (FFA). The increased content of liver FFA may be caused by increased uptake (from peripheral tissue, mainly adipose tissue, and to a lesser extent from dietary sources), increased de novo lipogenesis within the hepatocytes, or reduced utilization either through *β*-oxidation or via secretion (Figure 1).

The process of lipogenesis, by which acetyl-CoA is converted to fatty acids in the hepatocyte, is transcriptionally regulated by the membrane-bound transcription factor sterol regulatory element-binding protein-1c (SREBP-1c). SREBP-1c activates genes required for lipogenesis such as ATP-citrate lyase (ACL), acetyl-CoA carboxylase (ACC), fatty acid synthase (FAS), and stearoyl-CoA desaturase (SCD). Peroxisome proliferator-activated receptors (PPARs) are other major transcription factors that participate in regulation of fatty acid homeostasis by downstream gene regulation of targets responsible for lipid synthesis and oxidation [29, 30]. Increased hepatocyte lipogenesis is involved, at least in part, in the mechanism leading to amiodarone and tamoxifen hepatotoxicity [29, 31–34].

Another mechanism that may lead to increased hepatocyte FFA accumulation is decreased incorporation of FFA into very low-density lipoprotein (VLDL) or attenuated secretion of the latter. This mechanism was demonstrated in tamoxifen-induced steatosis [29]. The lipotoxic effect may not be merely a consequence of FFA accumulation but rather a more complex mechanism involving the lipid metabolism in hepatocytes as it appears that changes in non-high-density lipoprotein cholesterol may precede the onset of NAFLD [35].

Mitochondria play a pivotal role in lipid metabolism and ATP synthesis. As such they have been extensively investigated as targets for DILI. Studies have shown that drugs such as perhexiline, amiodarone, and tamoxifen can accumulate in mitochondria and interfere with the mitochondrial electron transport chain and *β*-oxidation, subsequently causing microvesicular steatosis and necrosis [36–38]. In fact, oxidative stress and lipid peroxidation mediated by production of reactive oxygen species (ROS) have been implicated as an important cause of DILI [39–46]. The concept that lack of antioxidant protection is involved in the progression of liver injury resulting from advanced inflammation and fibrosis is reinforced by studies demonstrating an ameliorating effect of several antioxidants on DIS [47–50].

## 5. NASH and Hepatotoxicity

Steatosis is an exceedingly common finding in liver biopsies and the possibility that NAFLD and not DIS is the cause of an underlying liver disease should always be considered in the context of the specific patient [19]. Features of the metabolic syndrome (MS) such as diabetes, insulin resistance, dyslipidemia, and obesity are closely related to the development of NAFLD [4]. It may be hypothesized that drugs such as glucocorticoids, tamoxifen, or VPA, that have metabolic effects and are known to induce weight gain, insulin resistance, and dyslipidemia, may precipitate steatosis through traditional NAFLD risk factors and not by direct hepatotoxicity [51–53].

The current concept of NAFLD and NASH is of a clinical spectrum of disease, resulting from a “multiple-hit” process [54, 55]. The mechanisms involved in the progression of simple steatosis to NASH are complex [56], preexisting hepatic steatosis may render the hepatocytes vulnerable to drug induced injury, and DIS may aggravate preexisting liver fat. For instance, recent data suggests that the steatotic liver is susceptible to oxidative stress induced by certain drugs [57]. Moreover, it appears that NASH causes significant changes in hepatic metabolism of drugs due to alternations in hepatic drug transporters and changes in drug pharmacology and pharmacokinetics, thus affecting their safety [58]. For example, it was shown that induction of NASH in rats leads to changes in expression and function of ATP-binding cassette (ABC) transporters responsible for the disposition of drugs, causing elevated expression of Abcc1-4, Abcb1, and Abcg2 transporters and mislocalization of Abcc2 and Abcb1 to the membrane thus rendering hepatocytes more susceptible to hepatocellular damage after administration of MTX [59]. Finally, assessment of genome-wide mRNA expression of genes responsible for drug metabolism and distribution in samples of human liver tissue revealed changes in global drug transport gene expression associated with progression from steatosis to NASH, suggesting that drug metabolism and toxicity may be altered in this disease [60].

The number of drugs associated with liver lipotoxicity is large. In the following section we describe several key drugs for which the evidence of steatosis induction is stronger and the mechanism of injury has been at least partially elucidated.

## 6. Antiarrhythmic Drugs

### 6.1. Amiodarone

Amiodarone chlorhydrate is a di-iodinated benzofuran derivative, class III antiarrhythmic drug, used for both ventricular and atrial arrhythmias. Amiodarone is associated with many adverse effects, such as thyroid dysfunction, corneal microdeposits, optic neuropathy, peripheral neuropathy, and pulmonary and hepatic toxicities [61].

Amiodarone hepatotoxicity usually occurs in two distinct clinical settings: the rapid intravenous infusion in acute settings, and the long-term chronic oral therapy. In the case of severe acute hepatitis following introduction of intravenous amiodarone, liver enzyme abnormalities occur within hours to days of treatment initiation and although most published cases were reversible after discontinuation of the treatment, acute liver failure and death have been reported. In some cases, a rechallenge with oral amiodarone was successful, leading some authors to ascribe the acute reaction to the effect of polysorbate 80 used as a solvent in the intravenous preparation [62–67].

With chronic amiodarone therapy, asymptomatic elevation of aminotransferases, usually up to 3 times the upper limit of normal (ULN), was reported in up to half of the patients, although in more recent studies the numbers are considerably lower [61, 68–71]. The latent period may vary from several weeks to a few years and in more than 90% of the patients it is over 90 days and although liver injury is usually reversible it may take weeks or even months for enzymes to normalize [71, 72]. Practice guidelines from the North American Society of Pacing and Electrophysiology/Heart Rhythm Society (NASPE/HRS) recommend measuring liver enzymes prior to initiation of treatment and repeated semiannual surveillance [73]. There is no data with regard to the progression of hepatic injury with continued therapy in patients with mildly elevated liver enzymes; however, a recent study showed that patients with mild aminotransferase elevation at baseline did not show a progressive rise in liver enzymes after a mean follow-up of 2 years of therapy [74].

Symptomatic hepatic dysfunction may occur in 1–3% of patients using amiodarone [61, 68–70]. In these steatosis cases, both macrovesicular and microvesicular are the most common pathological features. Steatohepatitis, hepatocytes ballooning, Mallory bodies, and fibrosis are also common. Other changes included nuclear unrest, acidophilic bodies, foam cells, glycogenated nuclei, and portal inflammation [75]. Amiodarone induced hepatic cirrhosis is also well documented [72, 76]. Unlike most other cases of DILI, liver damage may progress despite discontinuation of the drug [77]. Case reports of microvesicular steatosis and hepatocellular necrosis resembling Reye's syndrome have also been described [78] and rarely a granulomatous injury pattern may be observed [79].

Several mechanisms account for potential liver injury following amiodarone use. In this population of patients liver enzymes abnormality may be due to right-sided heart failure, MS, NASH, and other drugs [80]; however, a recent retrospective study concluded that the presence of MS or right-sided heart failure does not increase the incidence of amiodarone hepatotoxicity [70].

Amiodarone and its principal lipophilic metabolite, desethylamiodarone, are highly concentrated in the liver in a manner that appears to be related more closely to the total dose rather than to plasma concentrations [81]. On computerized tomography (CT) scans the iodine content of the drug causes significant increase in hepatic density; however, the clinical significance of this finding is questionable [80, 82]. Hepatic storage of amiodrone may cause phospholipidosis with a characteristic histopathological appearance of intracellular lamellar inclusion bodies formed by excessive accumulation of phospholipids [33]; this appears to be a generalized systemic effect of cationic amphiphilic compounds, independent from hepatic injury or steatohepatitis [75], and may result from direct inhibition of phospholipase or from the formation of nondegradable drug-phospholipid complexes [80]. Amiodarone induced phospholipidosis may also be explained by an indirect mechanism of upregulation of the fatty acid biosynthesis-related gene SCD, causing enhanced synthesis of phospholipids, and overexpression of lanosterol synthase (LSS), associated with cholesterol synthesis [33].

In animal models the hepatotoxic effect of amoidarone appears to be dose-dependent and consistent with increased production of cholesterol and accumulation of triglycerides in hepatocytes. In mouse studies, several genes were found to be regulated by amiodarone. Target enrichment of two nuclear receptors, androgen receptor and HNF4*α*, was observed, resulting in the increase of lipids in the liver. With amiodarone treatment, both PPAR*α* and PPAR*γ* targets were enriched suggesting a constant competition between increased lipid synthesis and the counter response of increased fatty acid oxidation [32]. In vitro study in human hepatoma HepaRG cells exposed to amiodarone resulted in vesicular steatosis characterized by an excessive accumulation of triglycerides together with the appearance of Oil Red O-stained lipid vesicles and overexpression of several genes involved in lipogenesis (SREBP1, FAS, and ACL) and droplet formation [33].

Another important mechanism, indicated by the microvesicular injury pattern, is mitochondrial dysfunction; indeed, amiodarone and its metabolite are concentrated in the hepatic mitochondria and have been shown to inhibit electron transport and uncoupled oxidative phosphorylation. In animal models amiodarone caused decreased mitochondrial *β*-oxidation and increased production of ROS. Thus, hepatotoxicity associated with amiodarone can at least, in part, be explained by mitochondrial *β*-oxidation of fatty acids and the subsequent production of microvesicular steatosis and induction of apoptosis and necrosis [36, 83].

### 6.2. Dronedarone

Dronedarone (Multaq), a new class III antiarrhythmic agent, is a noniodinated amiodarone derivative associated with fewer adverse effects and reduced toxicity [84]. Reports on the hepatotoxic effects of dronedarone have been controversial with abnormal liver function rates ranging from 0.5% to 12% in early clinical trials [85, 86]. Recently two cases of dronedarone induced acute liver failure requiring liver transplantation, occurring 4.5 and 6 months after therapy initiation, were reported, resulting in the US Food and Drug Administration recommendation for monitoring liver function parameters [87].

As in the case of amiodarone, inhibition of mitochondrial *β*-oxidation is a pivotal mechanism of dronedarone induced hepatotoxicity. In an in vivo study comparing mechanisms of hepatotoxicity of dronedarone and amiodarone, Felser et al. found that both caused cytotoxicity and apoptosis, in addition to reduced cellular ATP content compatible with impaired mitochondrial function. Both drugs caused uncoupling and inhibition of the mitochondrial respiratory chain and inhibition of mitochondrial *β*-oxidation leading to accumulation of ROS and intracellular lipids [88]. Interestingly, mice exposed to dronedarone demonstrated impairment of mitochondrial *β*-oxidation resulting from reduced activity of carnitine palmitoyltransferase I (CPT I) in liver mitochondria, without effecting the activity of the respiratory chain ex vivo [89].

## 7. Methotrexate

Methotrexate is a folate antagonist that is used in the treatment of malignancies and autoimmune diseases. While treatment of malignancies may involve administration of high dose (≥500 mg/m^2^) to low dose (<50 mg/m^2^) MTX over a short time period, treatment of autoimmune diseases usually involves low doses of MTX over long periods of time. The mechanism of action may differ according to the treatment dose. MTX competitively inhibits the enzyme dihydrofolate reductase (DHFR) interfering with purine and pyrimidine biosynthesis and consequently decreasing DNA and RNA synthesis.

MTX liver dysfunction is mostly associated with its chronic use in inflammatory disease, although acute hepatitis following high dose administration has been described [90]. Minor to moderate aminotransferase elevations were described in up to 50% of patients receiving chronic MTX therapy [21, 91]; however, recent studies have described a lower incidence of 6–24% [92–96]. Liver enzyme abnormalities under MTX treatment do not necessarily represent significant liver toxicity as they usually resolve with dose modification or drug discontinuation and may even normalize during the course of therapy [93]. Moreover, it was previously reported that 70–88% of patients that undergo liver biopsy exhibit normal histology or mild fatty changes [93, 97]. The estimated incidence of advanced pathologic changes, that is, significant fibrosis or cirrhosis, is 4-5% [97, 98]. The population burden of end-stage MTX-related liver disease is exceedingly small as it accounts for only 0.07% of liver transplantations in the United States [99].

**Figure 2:**
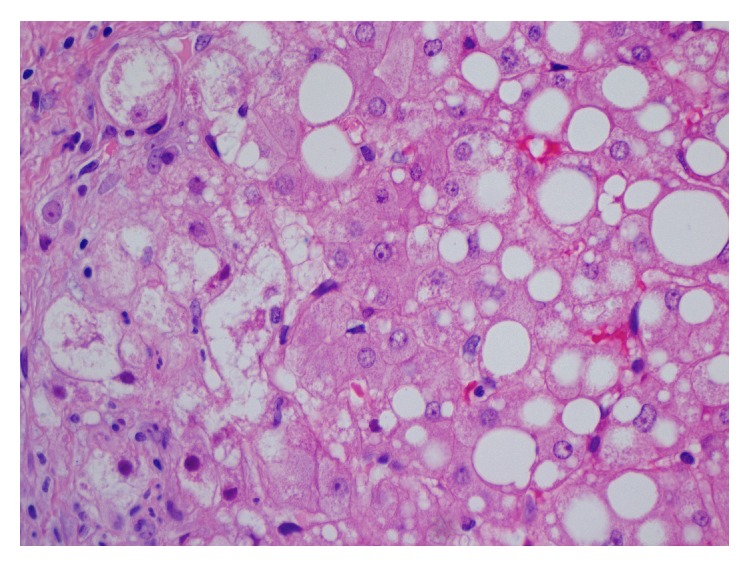
Liver histology of a patient with MTX induced hepatotoxicity demonstrating macro and microvesicular steatosis, hepatocellular ballooning, and Mallory hyaline bodies. H&E, original magnification ×400 (image courtesy of Dr. Eli Brazovsky, Tel-Aviv Medical Center, Israel).

The histological features of MTX induced liver injury consist of fatty changes of varying degrees, nuclear pleomorphism, hepatocyte necrosis, portal chronic inflammatory infiltrate, fibrosis that is typically pericellular and perivenular in early lesions with subsequent development of fibrous septa, and cirrhosis (Figure 2) [100].

Several risk factors have been associated with MTX induced hepatotoxicity including preexisting liver disease such as NAFLD, chronic hepatitis B or C (HBV, HCV), alcohol consumption, obesity, total cholesterol, and diabetes [92, 97].

The underlying mechanism of MTX hepatotoxicity is not fully understood. Oxidative stress has been implicated as an important cause through both increased production of ROS and decreased defense mechanisms. In vivo studies have demonstrated an increase of reactive oxygen metabolites such as myeloperoxidase (MPO), lipid peroxidation product malondialdehyde (MDA), and thiobarbituric acid reactive substances (TBARS) following MTX administration. Furthermore, a reduction in the levels of the antioxidant glutathione (GSH) was also demonstrated. However, increased levels of antioxidant enzymes such as catalase (CAT) and glutathione-S-transferase (GST) may suggest a possible adaptive mechanism of the liver [39–42]. In a murine model of steatosis following high-dose MTX exposure, the MTX-responsive genes were predominantly associated with the unfolded protein response and biosynthesis, catabolism, and transport of lipids and fatty acids [101].

## 8. Tamoxifen

Tamoxifen is a selective estrogen receptor modulator (SERM) with both agonist and antagonist effect in different tissues. Tamoxifen induced hepatotocixity manifesting as elevated liver enzymes may be found in up to 43% of the patients; however, normalization of liver enzymes is usually complete within 6 months of treatment discontinuation [51, 57]. Several forms of hepatotoxicity have been described in relationship with tamoxifen; however, its association with fatty liver is the most commonly encountered. Common findings in liver biopsies are mild to moderate steatohepatitis, macrovesicular steatosis, and rarely cirrhosis [51]. In a study of 1,105 breast cancer patients, NASH was documented in 2.2% with tamoxifen treatment increasing the odds of developing NASH by 8.2-fold [102]. In another study of 105 breast cancer patients treated with tamoxifen, hepatic steatosis was recognized in the majority of patients within the first 2 years of receiving adjuvant tamoxifen, with only half of the patients having increased transaminase levels [103]. In a prospective, randomized, double blind, placebo-controlled trial of 5,408 healthy women who underwent hysterectomies and were treated with tamoxifen chemoprevention, the hazard ratio for developing NASH was 2.0 compared with placebo, although the overall number of patients with NASH was considerably smaller in comparison with other reports (2%). Interestingly, the increases in NASH attributable to tamoxifen occurred in the first 2 years of therapy [57].

Factors associated with the development of tamoxifen induced liver injury are shared with traditional NAFLD risk factors and include glucose intolerance and diabetes, obesity, hyperlipidemia, and hypertension [51]. Genetic functional polymorphism may also influence individual susceptibility as in the case of cytochrome P450c17 [104].

In vitro HepG2 human hepatoma cell line treated with tamoxifen showed induced hepatocyte steatosis and increased hepatocyte triglyceride concentration. A possible mechanism of tamoxifen induced hepatic steatosis was via enhancement of fatty acid synthesis through the upregulation of SREBP-1c and its downstream lipogenesis target genes. Furthermore triglyceride accumulation stimulated microsomal triglyceride transfer protein (MTP) expression that was associated with VLDL assembly and secretion [29]. In vivo models also support these findings, demonstrating de novo fatty acid synthesis as the primary event leading to tamoxifen induced steatosis [31, 34].

The role of the mitochondria in tamoxifen induced hapatotoxicity is controversial. Rodent studies have indicated that tamoxifen accumulates in mitochondria where it impairs *β*-oxidation and respiration in part by inhibiting CPT I, the rate-limiting enzyme of mitochondrial fatty acid *β*-oxidation, and also inhibits topoisomerases thus progressively depleting hepatic mitochondrial DNA [37]. In contrast, in vitro study of HepG2 cells failed to demonstrate an effect of tamoxifen on the expression of CPT I [29]. Likewise, other murine models have also shown triglycerol secretion and fatty acid oxidation unaffected by tamoxifen [31, 34]. Oxidative stress appears to play a role in tamoxifen induced hepatotoxicity. Studies in rats exposed to tamoxifen resulted in depletion of liver reduced GSH and accumulation of oxidized glutathione and lipid peroxidation and also induced inhibition of hepatic activity of glutathione reductase (GR), glutathione peroxidase (GPx), superoxide dismutase (SOD), and CAT [43, 44].

## 9. Valproic Acid

Valproic acid (2-n-propylpentanoic acid) is widely used as a broad-spectrum antiepileptic drug. A wide range of adverse effects have been reported in connection with VPA, among which its potential hepatotoxicity is a major concern. Serum aminotransferases are commonly elevated in up to 44% of patients on VPA therapy; however, this elevation is generally mild (1–3x ULN) and does not involve a rise in serum bilirubin. Patients are mostly asymptomatic or suffer mild symptoms (malaise, lethargy, and anorexia). This presentation of VPA hepatotoxicity is frequently transient and resolves with dose reduction or drug discontinuation [105]. Incidence of fatal hepatotoxicity is relatively low and was reported between 1/37,000 and 1/5,000 cases, with the highest incidence found among children up to two years of age and with comedication [106–108]. This form of acute idiosyncratic liver injury is characterized by microvesicular steatosis and cell necrosis of varying degree. In general VPA-associated idiosyncratic hepatotoxicity has a delayed onset from weeks to months, and in some cases even years, following initial exposure [107, 109, 110].

In recent years the association between VPA treatment and NAFLD has been further studied and it is well recognized that long-term use of this drug can induce features of the MS consistent of substantial weight gain, insulin resistance, and lipid abnormalities [111]. Hepatic steatosis was reported in 61% of patients treated with VPA, based on abdominal ultrasound imaging [112]. Therefore, a possible mechanism of VPA induced steatosis could be via its metabolic effect promoting or worsening NAFLD and giving rise to macrovesicular steatosis and steatohepatitis [52, 53]. This notion is also supported by results from a rat model of high-fat diet and VPA administration, demonstrating increased hepatic steatosis and hepatotoxicity, as well as exacerbation of VPA-induced impairment of mitochondrial *β*-oxidation [113].

Other mechanisms of VPA induced hepatic steatosis are possibly related to the drug's metabolism, as VPA is primarily metabolized in the liver. One of the major metabolic pathways, accounting for 30–50% of VPA metabolism, is conjugation, predominantly with glucuronic acid but also other minor conjugation reactions with glutathione, carnitine, coenzyme A, and other amino acids, while the other main metabolic pathway is mitochondrial oxidative reactions, especially *β*-oxidation, which typically accounts for about 40% of its metabolism [109]. As in other conditions characterized by microvesicular steatosis, it appears that interfering with fatty acids mitochondrial *β*-oxidation plays a key role in VPA induced hepatic toxicity. The two main mechanisms involve mitochondrial dysfunction and oxidative stress. Formation of valproyl-CoA causes depletion of intramitochondrial CoA affecting fatty acid *β*-oxidation, impairment of ATP production, and inhibition of CPT1 as well as depletion of carnitine stores [114, 115]. An in vivo study of isolated rat hepatocytes showed that VPA induces significant increase in mitochondrial ROS production, disruption in electron transfer chain, and increase in lipid peroxidation, together with decreased levels of reduced glutathione. The induction of mitochondrial swelling, mitochondrial membrane potential collapse, and release of cytochrome c suggests that VPA may directly cause mitochondrial damage and activate the intrinsic death-signaling pathway [45, 46]. Interestingly, it was recently reported that administration of VPA results in a marked decrease in stellate cell activation, a pivotal step in the pathogenesis of liver fibrosis, and collagen deposition in both in vitro and in vivo mice models [116].

## 10. Nucleoside Reverse Transcriptase Inhibitors 

Antiretroviral therapy (ART) has markedly increased life expectancy in patients with the human immunodeficiency virus (HIV). ART regimens usually consist of NRTIs, usually as paired agents, with at least one other antiretroviral drug with a different mechanism of action such as a protease inhibitor (PI), and/or a nonnucleoside reverse transcriptase inhibitor (NNRTI).

Elevated liver enzymes are common among HIV patients receiving ART with an estimated prevalence of 20–40%. In patients with HBV/HCV coinfection it can be above 60%, with severe hepatotoxicity in about 10% of the patients [117]. All antiretroviral drugs have some risk of hepatotoxicity with each drug class associated with a characteristic pattern of injury, among which NRTIs have been most commonly associated with hepatic steatosis [118]. NRTIs include zidovudine (AZT), didanosine (ddI), stavudine (d4T), lamivudine (3TC), emtricitabine (FTC), abacavir (ABC), zalcitabine (ddC) which is no longer licensed, and tenofovir (TDF) [119]. In two large cross-sectional studies of monoinfected HIV patients receiving ART, the prevalence of NAFLD diagnosed by ultrasound or CT scan was 31–37%; risk factors associated with steatosis were increased transaminases, male sex, high body mass index (BMI) and waist circumference, serum lipid abnormalities, and NRTI exposure [120, 121].

Steatohepatits related to ART results from multiple mechanisms. The occurrence of NAFLD among these patients may be attributed to metabolic abnormalities commonly found among HIV patients receiving ART, in particular NRTI-PI combinations, and include subcutaneous lipoatrophy and accumulation of central fat, dyslipidemia, and insulin resistance [122]. Other mechanisms result from direct drug hepatotoxicity. NRTI interfere with viral reverse transcriptase enzyme activity but can also inhibit human DNA polymerase *γ*, the enzyme responsible for the replication of mitochondrial DNA (mtDNA); impairment of mitochondrial function causes the release of cellular lactate and defective liver fatty acid *β*-oxidation and may result in a spectrum of injuries ranging from potentially fatal hepatotoxicity and lactic acidosis to various degrees of hepatic marcovesicular and microvesicular steatosis [123, 124]. Mitochondrial injury was confirmed by ultrastructural studies with electron microscopy demonstrating mitochondrial abnormalities and an associated decreased expression of cytochrome oxidase (COX) subunits I, encoded by mtDNA [125, 126]. Another suggested mechanism of mitochondrial injury is inhibition of hepatocyte autophagic activity, demonstrated by in vivo exposure of hepatocytes to thymidine analogues zidovudine (ZDV) and d4T, leading to accumulation of dysfunctional mitochondria, increased ROS production, increased apoptosis, decreased proliferation, and increased intracellular lipid accumulation [127]. Hepatocyte lipid accumulation may also be a result of an alternative mtDNA-independent mechanism as demonstrated by in vivo study of murine hepatocytes exposed to d4T showing increased expression of SREBP-1c and reduction of MTP involved in lipid export and fatty acid oxidation inhibition without mtDNA depletion and lactate production [128].

## 11. Chemotherapy

Regimens containing FOLFOX (5-FU + folinic acid + oxaliplatin) and FOLFIRI (5-FU + folinic acid + IRI) are commonly used treatments for colorectal cancer [129]. Chemotherapy treatment is associated with histopathological liver changes, specifically sinusoidal obstruction syndrome associated with oxaliplatin, steatosis related to 5-FU, and CASH most commonly associated with IRI [130].

5-FU is a fluoropyrimidine antimetabolite that causes irreversible inhibition of thymidylate synthase. Although little is known about the mechanisms of hepatotoxicity associated with 5-FU, it is well recognized that mild and reversible hepatic enzymes abnormalities are common in patients treated with 5-FU and may be found in up to 40% of the patients [131]. Studies have shown that increased hepatic fat content may be found in 35–47% of patients receiving 5-FU, without correlation to chemotherapy dose or liver function tests [132, 133]. Predisposition to the development of steatosis may be associated with individual patient expression of genes involved in 5-FU metabolism, such as low level of dihydropyrimidine dehydrogenase (DPD) mRNA expression [134]. It has been suggested that other catabolites produced by DPD, such as fluoro-beta-alanine, remain in hepatocytes long after cessation of therapy and may saturate metabolic pathways reducing overall capacity to metabolize drugs and fat, leading to accumulation of intracellular lipids [135]. Furthermore it has been suggested that 5-FU is associated with collapse of the mitochondrial membrane leading to impaired *β*-oxidation and production of ROS [135].

IRI is a topoisomerase-1 inhibitor and a very active antineoplastic drug. Its use is associated with 5 times increased risk of CASH, found in 20% of treated patients, and 10 times increased 90-day mortality, specifically death from postoperative hepatic failure [130]. It has been suggested that mitochondrial DNA contains topoisomerase-1 highly homologous to the nuclear gene [136], which might serve as a target for IRI, causing mitochondrial dysfunction [135].

Other metabolic risk factors may play an important role in the development of steatosis or steatohepatitis in these patients, both as an underling condition or as part of the “multiple-hit” process. This notion is strengthened by findings of 208 patients with colorectal liver metastasis that underwent resection in whom BMI was the strongest predictor of steatosis and steatohepatitis, independent of the use of preoperative chemotherapy. Furthermore, among patients receiving preoperative chemotherapy, BMI was a predictor of chemotherapy liver injury with a 39% rate of steatosis or steatohepatitis among obese patients [137]. Interestingly, an analysis of 5853 patients undergoing liver resection for colorectal cancer without preoperative chemotherapy found a paradoxical advantage in overall survival and cancer-specific survival in patients with steatosis compared to those categorized as having “normal” liver, suggesting that excess body adiposity may have a survival protective effect [138].

The data regarding the prognostic effect of chemotherapy-induced steatosis or steatohepatitis is conflicting. While some studies have demonstrated reduced survival in patients with CASH [130], others challenge this perception demonstrating that preoperative chemotherapy-induced steatosis has no influence on overall or cancer-specific survival [139]. This issue is probably more complicated and depends on the ability to differentiate between different types of liver injury as demonstrated in an analysis of 119 patients where postoperative morbidity was associated with histological findings of parenchymal liver inflammation and steatohepatitis but not with isolated steatosis [140].

## 12. Summary

Hepatic steatosis is a rare form of DILI. DIS/DISH may present as macrovesicular steatosis, microvesicular steatosis, and steatohepatitis depending on the particular mechanism of a specific lipotoxic drug and may show variable latency prior to the occurrence of a clinically apparent injury. The pathogenesis of DISH from most drugs is complex involving major biological pathways of hepatocyte lipid metabolism. The relationship between DIS and “primary” NAFLD is of particular importance, as some drugs (such as glucocorticoids, tamoxifen, and VPA) clearly precipitate the induction of traditional NAFLD risk factors via their metabolic effect, while on the other hand it appears that preexisting steatosis may render the hepatocytes vulnerable to drug insults, may alter hepatic drug metabolism, and may eventually exacerbate existing damage.

The diagnosis of DIS/DISH requires a high index of suspicion because it represents a rare cause of steatosis. Thus a patient with DISH may erroneously be diagnosed as having “primary” NAFLD.

The potential reversibility of the initial liver injury, preventing the development of severe liver injury and ultimately liver fibrosis, underlines the importance of early recognition of this unique hepatotoxic effect. Better understanding of the molecular mechanisms involved and potential risk factors, as well as the intricate relationship with metabolic NAFLD, may offer potential clues in identifying patients more susceptible to this type of drug injury and better understanding of the mechanisms leading to metabolic steatohepatitis.

## Figures and Tables

**Table 1:** Main features of drug induced steatosis/steatohepatitis.

*Macrovesicular steatosis *	
Alcohol	
Amiodarone	
Chemotherapy (5-fluorouracil, tamoxifen, irinotecan, cisplatin, and asparaginase)	
Glucocorticoids	
Methotrexate	
Total parenteral nutrition	

*Steatohepatitis *	
Amiodarone	
Irinotecan	
Methotrexate	
Tamoxifen	

*Microvesicular steatosis *	
Aspirin (Reye syndrome)	
Cocaine	
Glucocorticoids	
Nonsteroidal anti-inflammatory drugs (NSAIDS): ibuprofen and naproxen	
Nucleoside reverse transcriptase inhibitors (NRTI)	
Tetracycline (Intravenous administration of high doses)	
Valproic acid	

## Abbreviations

DILI: Drug induced liver injury
NAFLD: Nonalcoholic fatty liver disease
NASH: Nonalcoholic steatohepatitis
HCC: Hepatocellular carcinoma
DIS: Drug induced steatosis
DISH: Drug induced steatohepatitis
CIOMS/RUCAM: Council for International Organizations of Medical Sciences/Roussel Uclaf Causality Assessment Method
NADRPS: Naranjo Adverse Drug Reactions Probability Scale
NAS: NALFD activity score
DILIN: Drug Induced Liver Injury Network
TPN: Total parenteral nutrition
MTX: Methotrexate
CASH: Chemotherapy associated steatosis or seatohepatitis
5-FU: 5-Fluorouracil
IRI: Irinotecan
VPA: Valproic acid
NRTI: Nucleoside reverse transcriptase inhibitors
NSAIDS: Nonsteroidal anti-inflammatory drugs
FFA: Fatty acids
SREBP-1c: Sterol regulatory element-binding protein-1c
ACL: ATP-citrate lyase
ACC: Acetyl-CoA carboxylase
FAS: Fatty acid synthase
SCD: Stearoyl-CoA desaturase
PPARs: Peroxisome proliferator-activated receptors
VLDL: Very low density lipoprotein
ROS: Reactive oxygen species
MS: Metabolic syndrome
ABC: ATP-binding cassette
ULN: Upper limit of normal
CT: Computerized tomography
NASPE/HRS: North American Society of Pacing & Electrophysiology/Heart Rhythm Society
LSS: Lanosterol synthase
CPT I: Carnitine-palmitoyl-transferase I
HBV: Hepatitis B virus
HCV: Hepatitis C virus
MPO: Myeloperoxidase
MDA: Malondialdehyde
TBARS: Thiobarbituric acid reactive substances
GSH: Glutathione
CAT: Catalase
GST: Glutathione-S-transferase
SERM: Selective estrogen receptor modulator
MTP: Triglyceride transfer protein
GR: Glutathione reductase
GPx: Glutathione peroxidase
SOD: Superoxide dismutase
ART: Antiretroviral therapy
HIV: Human immunodeficiency virus
PI: Protease inhibitor
NNRTI: Nonnucleoside reverse transcriptase inhibitor
AZT: Zidovudine
ddI: Didanosine
d4T: Stavudine
3TC: Lamivudine
FTC: Emtricitabine
ABC: Abacavir
ddC: Zalcitabine
TDF: Tenofovir
BMI: Body mass index
mtDNA: Mitochondrial DNA
COX: Cytochrome oxidase
ZDV: Zidovudine
DPD: Dihydropyrimidine dehydrogenase.
